# Scoring System-Based Approach for Positive Intracoronary Acetylcholine Provocation Tests: The Original and Modified ABCD Scores

**DOI:** 10.1016/j.jacadv.2025.101790

**Published:** 2025-05-14

**Authors:** Yoshiyuki Ohnaga, Yuichi Saito, Yuichiro Mori, Ken Kato, Kazuya Tateishi, Hideki Kitahara, Yoshio Kobayashi

**Affiliations:** a Department of Cardiovascular Medicine, Chiba University Hospital, Chiba, Japan; b Department of Human Health Sciences, Graduate School of Medicine, Kyoto University, Kyoto, Japan

**Keywords:** ischemic heart disease, vasospastic angina, risk score

## Abstract

**Background:**

Although intracoronary acetylcholine (ACh) provocation testing is a guideline-recommended invasive standard for the diagnosis of vasospastic angina (VSA), ACh tests are largely underused in clinical practice globally. Recently, the ABCD score, consisting of clinical presentation, myocardial bridge, C-reactive protein, and dyslipidemia, was developed to predict positive ACh test results.

**Objectives:**

The authors aimed to externally validate the diagnostic ability of the score and attempted to improve the predictivity for identifying patients with VSA.

**Methods:**

From May 2012 to September 2023, a total of 723 patients undergoing ACh provocation tests for diagnosing VSA were included. The original ABCD score was calculated according to the predefined criteria, and the modified ABCD score was internally developed to improve the diagnostic accuracy. The positive ACh provocation test (ie, VSA) was defined as a significant angiographic vasospasm accompanied by chest pain and/or ischemic electrocardiographic changes.

**Results:**

Of the 723 patients, 383 (53.0%) had positive ACh provocation test results. The receiver-operating characteristics curve analysis indicated that the original ABCD score was significantly predictive of positive ACh tests. Using best cutoff values on receiver-operating characteristics curve analyses, we developed the modified ABCD score, which was simpler than the original score. The modified rather than original ABCD score had better diagnostic ability for positive ACh test results (area under the curve 0.65 vs 0.55; *P* < 0.001).

**Conclusions:**

The original ABCD score was predictive of VSA in this external validation study with modest diagnostic accuracy, while the modified ABCD score achieved better predictivity for identifying patients with VSA.

Vasospastic angina (VSA) is a subset of ischemic heart disease and is a coronary vasomotor disorder that can induce myocardial infarction (MI) and life-threatening ventricular arrhythmia.^1^^,^^2^ The accurate diagnosis of VSA is clinically relevant to provide appropriate therapeutic strategies for better quality of life and preventing cardiovascular events.^3^^,^^4^ Intracoronary acetylcholine (ACh) provocation testing is a guideline-recommended invasive standard for the diagnosis of VSA,^5^^,^^6^ but is currently underused in daily clinical practice globally. In previous literature, several factors were reported to be associated with the positive diagnosis of VSA, such as genetic variants,^7^^,^^8^ older age,^9^ current smoking,^10^ dyslipidemia,^9^ a higher serum uric acid level,^11^ and MI with nonobstructive coronary arteries (MINOCA) presentation (ie, positive cardiac troponin).^12^ In this context, Rinaldi et al^13^ recently developed a risk-scoring system, the ABCD score, consisting of MINOCA presentation, myocardial bridge (MB), C-reactive protein (CRP), and dyslipidemia, to predict positive ACh test results, which has not been externally validated yet. In the present study, we aimed to test the diagnostic ability of the original ABCD score and attempted to improve the predictivity for identifying patients with VSA.

## Methods

### Study population

**Figure 1:**
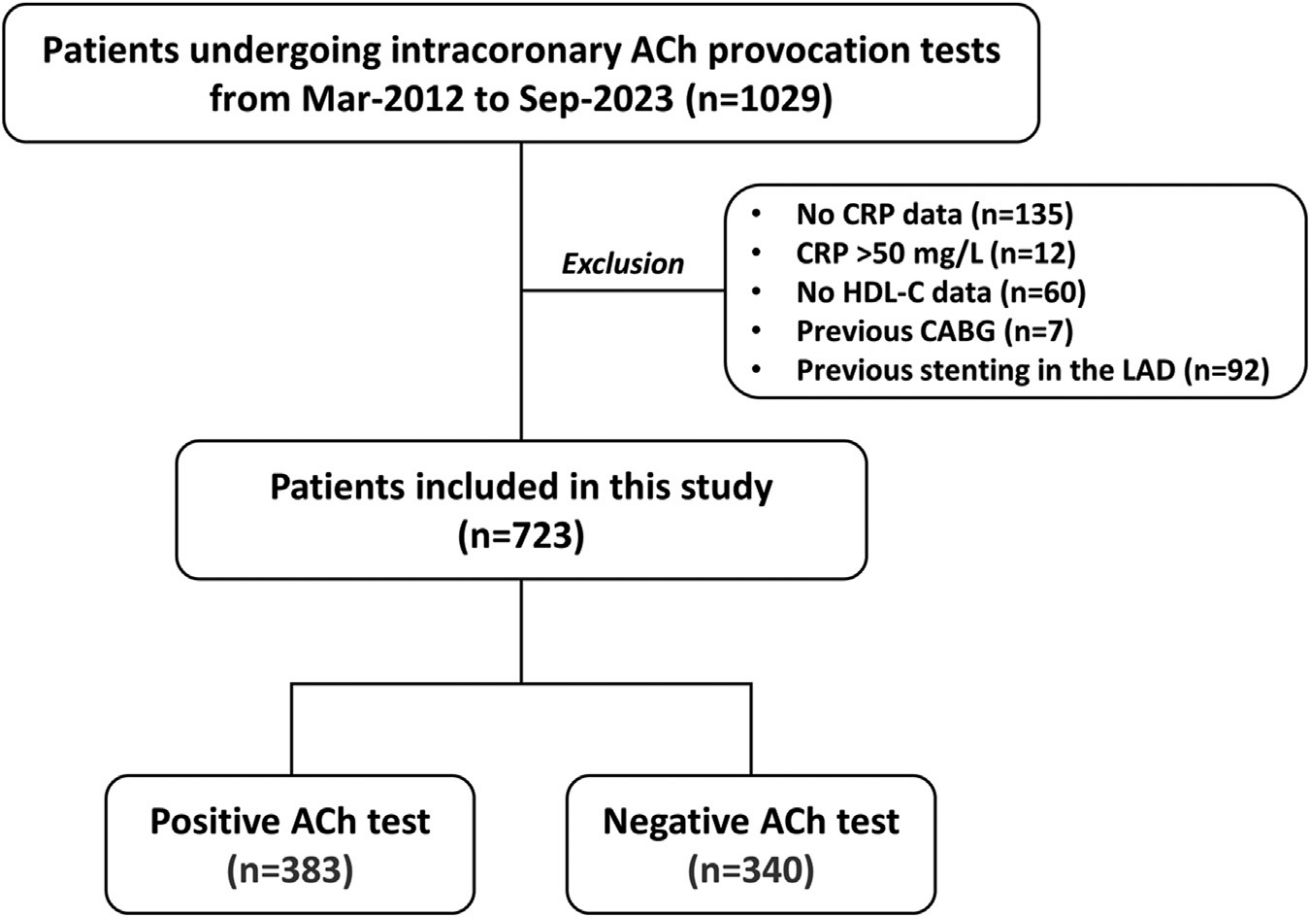
Study Flow ACh = acetylcholine; CABG = coronary artery bypass grafting; CRP = C-reactive protein; HDL-C = high-density lipoprotein cholesterol; LAD = left anterior descending artery.

From May 2012 to September 2023, a total of 1,029 patients underwent intracoronary ACh provocation testing for diagnosing VSA at Chiba University Hospital. Patients with missing data on CRP and high-density lipoprotein cholesterol (HDL-C) and those who had a history of coronary stenting in the left anterior descending artery and coronary artery bypass grafting were excluded (Figure 1). Additionally, patients with CRP >50 mg/L were further excluded to avoid an acute infectious disease or chronic systemic inflammatory conditions (Figure 1).^14^ Thus, 723 patients were included in this study. This study was done in accordance with the Declaration of Helsinki and was approved by the ethics committee of Chiba University Graduate School of Medicine. Informed consent was obtained in an opt-out manner.

### Coronary angiography and acetylcholine provocation test

Intracoronary ACh provocation tests were performed based on the Japanese guidelines in a clinical setting,^5^^,^^15^ as reported previously.^16^, ^17^, ^18^, ^19^ In brief, vasodilators, including calcium-channel blockers and long-acting nitrates, were discontinued ≥48 hours before the examination in elective cases, except for short-acting sublingual nitroglycerin, as needed. After control angiography in the right and left coronary arteries, a temporary pacing electrode was inserted in the right ventricle. Then, ACh was injected in incremental doses of 20, 50, and 100 μg into the left coronary artery initially and 20 and 50 μg into the right coronary artery subsequently, over 20 seconds at each administration. Coronary angiography was performed 1 minute after each ACh injection. Finally, after intracoronary administration of isosorbide dinitrate (1-2 mg), coronary angiography was performed. Angiographic coronary artery spasm was defined as epicardial coronary diameter reduction ≥90% in comparison with the coronary diameter following intracoronary isosorbide dinitrate injection. The positive diagnosis of intracoronary ACh provocation test was defined as angiographic coronary artery vasospasm accompanied by chest pain and/or ischemic electrocardiographic change.^5^

MB was assessed by 2 independent cardiologists on coronary angiography after isosorbide dinitrate injection. Angiographic MB was identified as the coronary vessel narrowing in systole that was more pronounced than in neighboring normal references, with partial or complete decompression during diastole (ie, milking effect).^20^ Quantitative coronary angiography analysis was performed to assess the severity of MB using QAngio XA (Version 7.1, Medis Medical Imaging System BV).^21^ The percent systolic compression and length of MB were evaluated based on quantitative coronary angiography. The presence of epicardial coronary artery disease was defined as the percentage of diameter stenosis >50% on visual assessment.

### The original and modified ABCD scores

**Figure 2:**
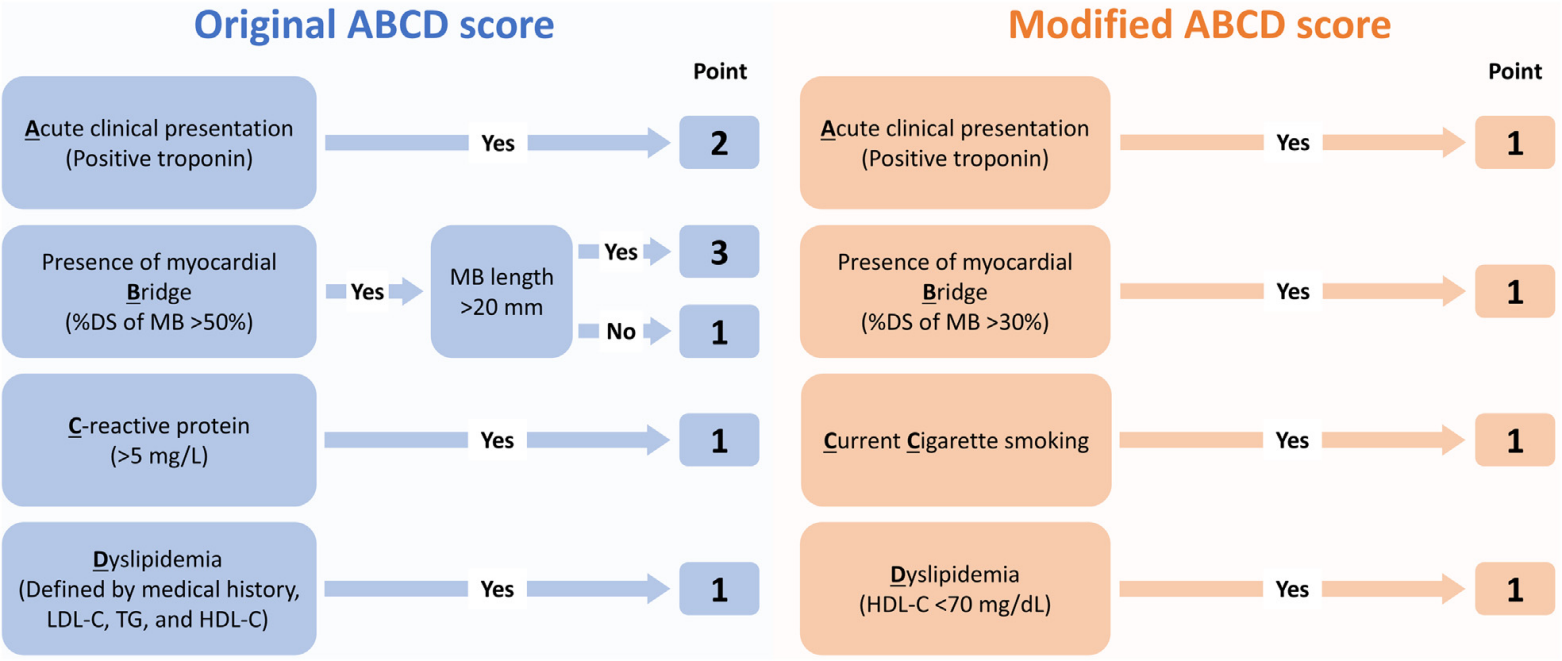
Original and Modified ABCD Scores If each variable is present, assigned points apply to the original and modified ABCD scores. The scores range from 0 to 7 in the original ABCD score and from 0 to 4 in the modified ABCD score. DS = diameter stenosis; LDL-C = low-density lipoprotein cholesterol; MB = myocardial bridge; TG = triglyceride; other abbreviation as in Figure 1.

The original ABCD score is a risk-predicting system for positive ACh test results, consisting of 4 variables (acute clinical presentation, MB, CRP, and dyslipidemia) (Figure 2).^13^ In the original ABCD score, acute clinical presentation, defined as positive cardiac troponin (ie, MINOCA), was assigned a score of 2. MB was angiographically defined as a >50% reduction in the luminal diameter of the coronary artery in systole, and a score of 3 was assigned if the MB length was >20 mm, and 1 point was assigned if the MB length was ≤20 mm (Figure 2). When a CRP level >5 mg/L, a score of 1 was assigned. Dyslipidemia was defined as a medical history of dyslipidemia, elevated levels of low-density lipoprotein cholesterol (LDL-C) (≥130 mg/dL) or triglycerides (≥175 mg/dL), or a reduced HDL-C level (<40 mg/dL in men or <50 mg/-dL in women). One point was assigned to patients with dyslipidemia.^13^ The original ABCD score showed good predictive ability for positive ACh test results in the proof-of-concept study with the area under the curve (AUC) of 0.703 in the derivation cohort and 0.705 in the internal validation cohort.^13^ The higher original ABCD score (range, 0-7) indicates a higher likelihood of positive ACh test results. In particular, patients with the ABCD score ≥4 points were diagnosed as having VSA in 94.3% in the original report.^13^ To improve diagnostic accuracy, we developed the modified ABCD score using best cutoff values on receiver-operating characteristics (ROC) curve analyses on percent MB compression and levels of HDL-C. All blood examinations were assessed at baseline. CRP was evaluated using a high-sensitivity assay (CRP II Latex X2, Denka-Seiken).^22^

### Statistical analysis

Statistical analysis was conducted with R statistical software package version 4.2.0 (The R Foundation for Statistical Computing). Data are expressed as mean ± SD, median (10th-90th percentiles), or frequency with percentage, as appropriate. Continuous variables were compared using Student's *t*-test or Mann-Whitney *U* test, and categorical variables were evaluated with the chi-squared test or Fisher exact test. Pearson's correlation coefficient was employed to assess a correlation between the length and percentage of compression of MB. The ROC curve analysis was performed to calculate the AUC based on a positive ACh test and evaluated the diagnostic ability of the original ABCD score. For the development of the modified ABCD score, an internally derived scoring system from the present study, multivariable analysis with a logistic regression model was performed to identify factors associated with positive ACh test results. Variables significantly associated with positive ACh testing on univariable analyses (*P* < 0.05) and the components of the original ABCD score were included in the multivariable model. First, continuous variables in the modified ABCD score were incorporated as being continuous. Then, the best cutoff values, which were established based on Youden's index for each variable to find the values corresponding to the maximum average sensitivity and specificity, were applied to the continuous variables in the modified ABCD score. In addition, the approximate numbers to the best cutoff values were arbitrarily used for developing the modified ABCD score. The diagnostic ability of models of the modified ABCD scores (with the continuous variables, best cutoff values, and approximate numbers) was evaluated and internally validated as described below. The AUCs between the original and modified ABCD scores were compared using the Delong method.

We conducted internal validation using Harrell's bias correction method for optimism adjustment with 10,000 bootstrap samples,^23^^,^^24^ applied to AUCs of the 3 differently developed modified ABCD scores: 1) MINOCA presentation, percentage of MB compression (as a continuous variable), current smoking, and HDL-C level (as a continuous variable); 2) MINOCA presentation, percentage of MB compression >26.8% (the best cutoff value on the ROC curve analysis), current smoking, and HDL-C level <71 mg/dL (the best cutoff value); and 3) MINOCA presentation, percentage of MB compression >30% (approximate number to the best cutoff value), current smoking, and HDL-C level <70 mg/dL (approximate number). In each sample, we refitted models, estimated AUCs for the sample itself and the original data, and calculated the difference between their AUCs as optimism. Optimism-adjusted AUCs were calculated by subtracting the mean optimism from the original AUCs. The 95% CIs were derived from the 2.5th and 97.5th percentiles of the bootstrap distribution. The analysis was carried out based on the TRIPOD-AI (Transparent Reporting of a Multivariable Prediction Model for Individual Prognosis Or Diagnosis-Artificial Intelligence) guidelines by a biostatistician.^25^ Because this study was performed in a retrospective manner, sample size calculation and study registration were not done. The data will be available upon a reasonable request. The present study had no patient and public involvement. There were no missing data on the components of the risk scores. A value of *P* < 0.05 was considered statistically significant.

## Results

Of the 723 patients, ACh provocation tests were positive in 383 (53.0%). Patients with positive ACh tests were more likely to be men (57.7% vs 45.9%; *P* = 0.002) and current smokers (24.7% vs 12.5%; *P* < 0.001) as compared to those with negative test results (Table 1). The rate of MINOCA presentation was higher in patients with positive ACh test results than their counterparts without statistical significance (10.2% vs 7.1%; *P* = 0.148). Levels of HDL-C were significantly lower in the positive ACh group than in the negative ACh group (59.4 ± 17.0 vs 64.9 ± 19.7 mg/dL; *P* < 0.001), while CRP levels did not differ significantly between the 2 groups (Table 1). MB was more frequently observed in patients with positive ACh tests (43.6% vs 24.4%; *P* < 0.001), with a higher percentage of compression (39.5% ± 16.7% vs 30.0% ± 14.4%; *P* < 0.001) and longer bridged length (18.5 ± 8.1 vs 15.2 ± 7.9 mm; *P* = 0.002) (Table 1). The bridged length was positively correlated with the percentage of compression of MB (r = 0.40; 95% CI: 0.29-0.50; *P* < 0.001) (Supplemental Figure 1).

**Table 1:** Baseline Characteristics

	All (N = 723)	Positive ACh Test (n = 383)	Negative ACh Test (n = 340)	*P* Value
Age (y)	63.7 ± 12.9	63.7 ± 12.6	63.8 ± 13.3	0.903
Men	377 (52.1%)	221 (57.7%)	156 (45.9%)	0.002
BMI (kg/m^2^)	23.9 ± 4.3	24.1 ± 3.8	23.7 ± 4.7	0.222
Hypertension	418 (57.8%)	209 (54.6%)	209 (61.5%)	0.070
Diabetes	135 (18.7%)	67 (17.5%)	68 (20.0%)	0.392
Dyslipidemia	530 (73.3%)	284 (74.2%)	246 (72.4%)	0.614
Current cigarette smoking	135 (18.7%)	93 (24.7%)	42 (12.5%)	<0.001
Previous MI	46 (6.5%)	28 (7.4%)	18 (5.4%)	0.359
Previous PCI	62 (8.6%)	37 (9.7%)	25 (7.4%)	0.289
MINOCA presentation	63 (8.7%)	39 (10.2%)	24 (7.1%)	0.148
Epicardial CAD	153 (21.2%)	85 (22.2%)	68 (20.0%)	0.523
Laboratory data				
eGFR (mL/min/1.73 m^2^)	73.1 ± 19.0	73.6 ± 18.2	72.6 ± 20.0	0.495
LDL-C (mg/dL)	113.3 ± 31.6	114.4 ± 32.5	112.0 ± 30.6	0.310
Triglycerides (mg/dL)	140.6 ± 97.8	141.4 ± 101.3	139.7 ± 93.8	0.821
HDL-C (mg/dL)	62.0 ± 18.5	59.4 ± 17.0	64.9 ± 19.7	<0.001
Uric acid (mg/dL)	5.3 ± 1.5	5.4 ± 1.5	5.2 ± 1.5	0.065
CRP (mg/L)	0.21 ± 0.47	0.23 ± 0.52	0.20 ± 0.42	0.414
Angiographic data				
Presence of MB	250 (34.6%)	167 (43.6%)	83 (24.4%)	<0.001
DS of MB (%)	36.4 ± 16.6	39.5 ± 16.7	30.0 ± 14.4	<0.001
MB length (mm)	17.4 ± 8.2	18.5 ± 8.1	15.2 ± 7.9	0.002
Epicardial CAD	153 (21.2%)	85 (22.2%)	68 (20.0%)	0.523
Medical treatment				
Antiplatelet agent	175 (24.2%)	106 (27.7%)	69 (20.4%)	0.024
Anticoagulant	56 (7.7%)	27 (7.1%)	29 (8.6%)	0.488
β-blocker	88 (12.2%)	45 (11.8%)	43 (12.7%)	0.733
Calcium-channel blocker	317 (43.8%)	157 (41%)	160 (47.1%)	0.115
ACE-I or ARB	224 (31.0%)	120 (31.4%)	104 (30.7%)	0.872
Diuretic	63 (8.7%)	28 (7.3%)	35 (10.3%)	0.186
Statin	242 (33.5%)	132 (34.6%)	110 (32.4%)	0.581
Nitrate	123 (17.0%)	81 (21.2%)	42 (12.4%)	0.002
Nicorandil	57 (7.9%)	36 (9.4%)	21 (6.2%)	0.128

Values are mean ± SD or n (%).

ACE-I = angiotensin-converting enzyme inhibitor; ACh = acetylcholine; ARB = angiotensin receptor II blocker; BMI = body mass index; CAD = coronary artery disease; CRP = C-reactive protein; DS = diameter stenosis; eGFR = estimated glomerular filtration rate; HDL-C = high-density lipoprotein cholesterol; LDL-C = low-density lipoprotein cholesterol; MB = myocardial bridge; MI = myocardial infarction: MINOCA = myocardial infarction with nonobstructive coronary arteries; PCI = percutaneous coronary intervention.

Overall, the median (10th-90th percentiles) original ABCD score was 1 (0-3) in the positive ACh group and 1 (0-2) in the negative ACh group (*P* = 0.010). In the ROC curve analysis, the original ABCD score had only modest diagnostic ability for positive ACh test results (AUC: 0.549; 95% CI: 0.513-0.586, best cutoff value 2 points; *P* < 0.001). With the best cutoff value, sensitivity, specificity, positive and negative predictive values, and positive and negative likelihood ratios of the original ABCD score were 0.24, 0.86, 0.65, 0.50, 1.67, and 0.89, respectively.

The multivariable analysis identified MB, current cigarette smoking, and HDL-C levels as factors significantly related to positive ACh test results, while MINOCA presentation tended to be associated with positive ACh testing (Supplemental Table 1). For the development of an improved risk-predicting score, we incorporated current cigarette smoking instead of CRP in the modified ABCD score because of the lack of statistical significance of CRP in the multivariable analysis (Supplemental Table 1). With a logistic regression model using these 4 variables (the percentage of MB compression and HDL-C levels were used as continuous variables), the ROC curve analysis showed an AUC of 0.674 (95% CI: 0.635-0.712) (Supplemental Table 2). The bootstrap-based internal validation confirmed a similar AUC (0.669; 95% CI: 0.632-0.706) (Supplemental Table 2). For clinical utility, we developed the modified ABCD score with dichotomizing continuous variables (ie, the percentage of MB compression and HDL-C levels) and assigning 1 point for each component. Based on the ROC curve analyses, the best cutoff values of the percentage of MB compression and HDL-C were 26.8% and 71 mg/dL, respectively (Supplemental Figure 2). Approximate numbers of these cutoff values (30% for the percentage of MB compression and 70 mg/dL for HDL-C) were adopted (Figure 2). The modified ABCD score ranged from 0 to 4 points. Higher values indicate a higher likelihood of positive ACh test results. The median (10th-90th percentiles) modified ABCD score was 1 (0-2) in the positive ACh group and 1 (0-2) in the negative ACh group (*P* < 0.001). The ROC curve analysis showed better diagnostic accuracy for positive ACh test results in the modified ABCD score (AUC: 0.650; 95% CI: 0.613-0.686; *P* < 0.001, best cutoff value 2 points) than in the original ABCD score (*P* < 0.001) (Central Illustration). The bootstrap-based internal validation confirmed nearly identical results (adjusted AUC: 0.649; 95% CI: 0.615-0.685) (Supplemental Table 2). The distribution of the original and modified ABCD scores is displayed in Central Illustrations, illustrating better stratification of positive ACh test results in the modified ABCD score.

**Central Illustration:**
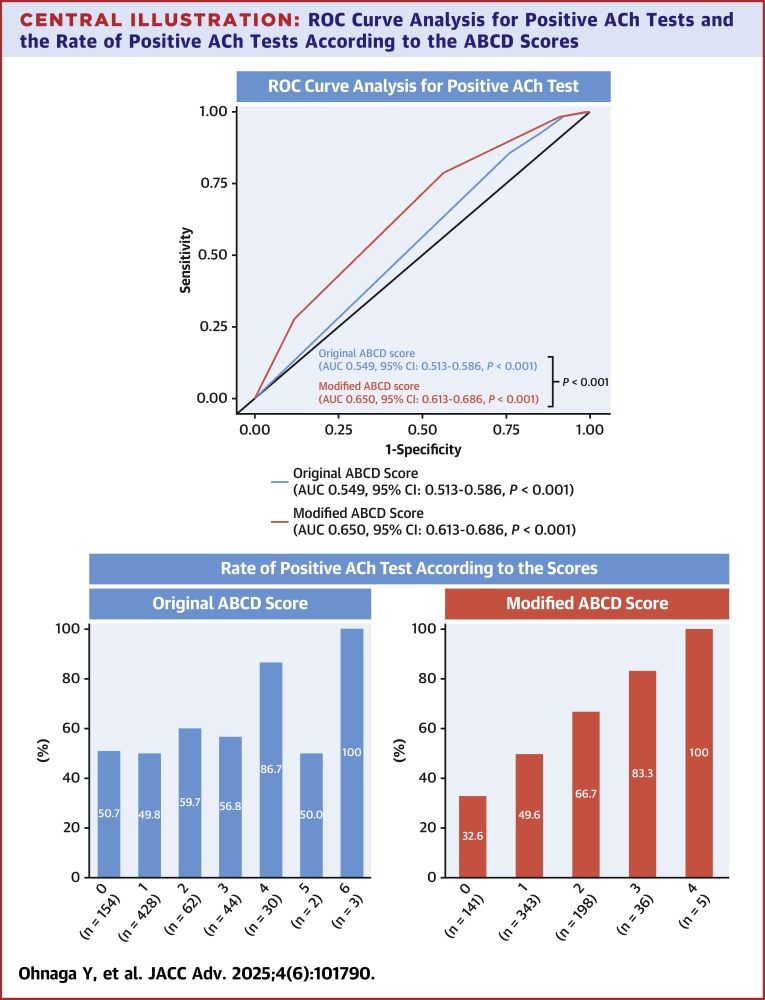
ROC Curve Analysis for Positive ACh Tests and the Rate of Positive ACh Tests According to the ABCD Scores For estimating positive ACh test results (ie, vasospastic angina), the modified rather than original ABCD score had better diagnostic ability. The modified ABCD score resulted in better stratification of positive ACh test results than the original ABCD score. AUC = area under the curve; ROC = receiver-operating characteristic; other abbreviation as in Figure 1.

When using the best cutoff values for the percentage of MB and HDL-C (ie, 26.8% and 71 mg/dL), the AUC of the modified ABCD score (0.656; 95% CI: 0.620-0.693; *P* < 0.001) was similar to that with the approximate numbers (ie, 30% and 70 mg/dL) (AUC: 0.650) (Central Illustration, Supplemental Figure 3, Supplemental Table 2). When omitting MINOCA presentation from the modified ABCD score because of the lack of statistical significance on the multivariable model (Supplemental Table 1), namely the “modified BCD score,” the AUC for positive ACh test results was 0.648 (95% CI: 0.612-0.684, best cutoff value 2 points; *P* < 0.001), which was similar to that of the modified ABCD score (Supplemental Figure 4).

## Discussion

The present study demonstrated that in patients undergoing intracoronary ACh provocation testing for the diagnosis of VSA, the calculation of the original ABCD score was clinically feasible. Although the higher original ABCD score was associated with a higher likelihood of positive ACh tests, the diagnostic ability was modest. The modified ABCD score that we developed using different definitions and cutoff values is simple and had better predictivity for VSA. The original and modified ABCD scores may be useful in identifying patients likely to experience a positive response to ACh provocation testing.

### Predictors of positive acetylcholine provocation testing

VSA is a major subset of ischemic heart disease, particularly in ischemia with nonobstructive coronary artery disease and MINOCA.^5^ The accurate diagnosis of VSA is relevant for tailoring therapeutic approaches, and the current guidelines recommend intracoronary ACh provocation testing.^5^^,^^6^ However, the diagnostic protocol varies widely among studies and institutions, and the penetration rate of ACh testing in daily practice is low globally.^26^ Thus, the prediction of VSA diagnosis without the implementation of invasive provocation testing would be clinically helpful, even after coronary angiography is performed. From a genetic viewpoint, Japanese researchers reported that deficient activity of the variant aldehyde dehydrogenase 2 and thereby alcohol flushing syndrome were more frequently found in patients with positive rather than negative ACh provocation tests, potentially supporting the higher prevalence of VSA in East Asia than in Western countries.^7^ Another recent case-control genome-wide association study in Japan also identified variants at the *RNF213* locus as factors associated with VSA.^8^ Although mechanistically important, genetic testing is usually challenging in real-world practice. From a clinical point of view, several variables have been identified as predictors of positive ACh provocation testing. Male gender, older age, and current smoking may be associated with a positive diagnosis of ACh tests.^9^^,^^10^ Some metabolic factors including dyslipidemia and an elevated serum uric acid level are reportedly related to the VSA diagnosis.^9^^,^^11^ Angiographically assessed MB is another predictor of positive ACh provocation testing in several previous reports.^20^^,^^27^, ^28^, ^29^ In this context, a scoring system-based approach for identifying patients with positive intracoronary ACh provocation testing, the original ABCD score, was recently developed.^13^

### Original and modified ABCD scores

The original ABCD score reported by Rinaldi et al^13^ includes 4 components, such as acute clinical presentation (ie, MINOCA), MB, CRP, and dyslipidemia. These 4 variables are readily available in a clinical practice setting when coronary angiography is performed. Indeed, the original ABCD score was assessable in more than 70% of cases in the present retrospective study. In their proof-of-concept study, the original ABCD score had good diagnostic ability (AUC: 0.70) for predicting positive intracoronary ACh provocation testing,^13^ although the external validation has not yet been performed. In the present study, the original ABCD score was significantly predictive of VSA, but the diagnostic accuracy was unsatisfactory with AUC <0.6 (ie, 0.55).^30^ Hence, we attempted to modify the ABCD score to be simpler and to have better diagnostic ability. Interestingly, the original ABCD score includes MINOCA presentation (positive cardiac troponin) as a component. The rate of MINOCA presentation was nonsignificantly higher in the positive ACh test group than the counterpart in the present study. Considering the clinical relevance of MINOCA, this variable may be important. However, whether the MINOCA presentation deserves relatively higher points to be assigned (ie, 2 points in the original ABCD score) is uncertain. MB is reportedly a strong predictor of VSA,^20^^,^^27^, ^28^, ^29^ as shown in the present study. Although the best cutoff value of the percentage of angiographical MB compression remains to be established, our previous study indicated 33.7% as the threshold,^20^ which is in line with the present study results. Given that the percentage of MB compression is correlated with the length of bridged segment, the former can be simply used for evaluating MB. Because inflammation is one of the underlying mechanisms of VSA,^31^ an elevated CRP level might be intuitively associated with positive ACh testing. However, CRP levels did not differ significantly between the 2 groups in the present study, while patients with positive ACh tests were more likely to be current smokers, which is in line with the previous literature.^5^^,^^10^ Although the male gender may be another variable to be incorporated, it may be confounded by the smoking habit.^10^ Therefore, we included current cigarette smoking in the modified ABCD score instead of CRP levels. Dyslipidemia was evaluated with a combination of medical history of dyslipidemia and laboratory data including levels of LDL-C, triglycerides, and HDL-C in the original study, among which HDL-C may be a key determinant of VSA.^10^^,^^32^ In the present study, the prevalence of dyslipidemia and the levels of LDL-C and triglyceride did not differ between patients with and without positive ACh provocation tests, while the HDL-C level was significantly lower in the positive test group. These laboratory findings beyond the presence of dyslipidemia were unavailable in the original study, and thus, we included HDL-C levels as the only determinant for dyslipidemia in the modified ABCD score for simplicity. Even though the modified ABCD score achieved better diagnostic accuracy for positive ACh testing as compared to the original score in the present study, the AUC on ROC curve analysis was 0.650, indicating still unsatisfactory diagnostic ability for clinical use.^30^ Further studies are warranted to externally validate the modified ABCD score and to improve the predictivity, as we did in the present study.

### Study limitations

The present study has some limitations. This is a single-center, retrospective study with a moderate sample size. From an ethnic perspective, whether the present results can be extrapolated to other regions than East Asia is unclear. Because MB is predominantly located in the left anterior descending artery,^33^ we excluded patients with previous coronary stenting in the vessel. MB was evaluated on coronary angiography in this study with no other diagnostic modalities (eg, intravascular ultrasound) and stress testing (eg, dobutamine). Although we focused on the ABCD score in the present study for the purpose of external validation, comprehensive risk assessment using artificial intelligence may be promising for future research.^34^ Even though we incorporated several clinical, laboratory, and angiographic data to develop the modified ABCD score, there may be plenty of other potential covariates for modeling. From a perspective of clinical applicability and practicality, one point was assigned to each variable with approximate numbers to simplify the scoring system.

## Conclusions

In the present external validation study, the original ABCD score had only modest diagnostic ability for positive ACh test results. The modified ABCD score was simpler and had better diagnostic ability for positive ACh testing than the original one. Further studies are needed to externally validate the modified ABCD score and to improve the diagnostic accuracy of risk-scoring models in patients suspected of VSA.

## Funding support and author disclosures

The authors have reported that they have no relationships relevant to the contents of this paper to disclose.

Perspectives **COMPETENCY IN MEDICAL KNOWLEDGE:** The original ABCD score, consisting of MINOCA presentation, MB, CRP, and dyslipidemia, was developed to predict positive ACh test results, despite the lack of external validation. The present study confirmed the significant predictivity of the ABCD score for a positive response to ACh provocation testing.

**TRANSLATIONAL OUTLOOK:** We developed the modified ABCD score, which is simpler and has better diagnostic ability than the original one. Although further studies are needed, the original and modified ABCD scores may aid in clinical decision-making in patients suspected of VSA.
