# Supplementary material for: Scoring System-Based Approach for Positive Intracoronary Acetylcholine Provocation Tests: The Original and Modified ABCD Scores
Source: JACC Adv. 2025 May 14;4(6):101790. doi: 10.1016/j.jacadv.2025.101790 (PMC12142498; doi:10.1016/j.jacadv.2025.101790)
Supplement: Supplementary data [file mmc1.pdf]

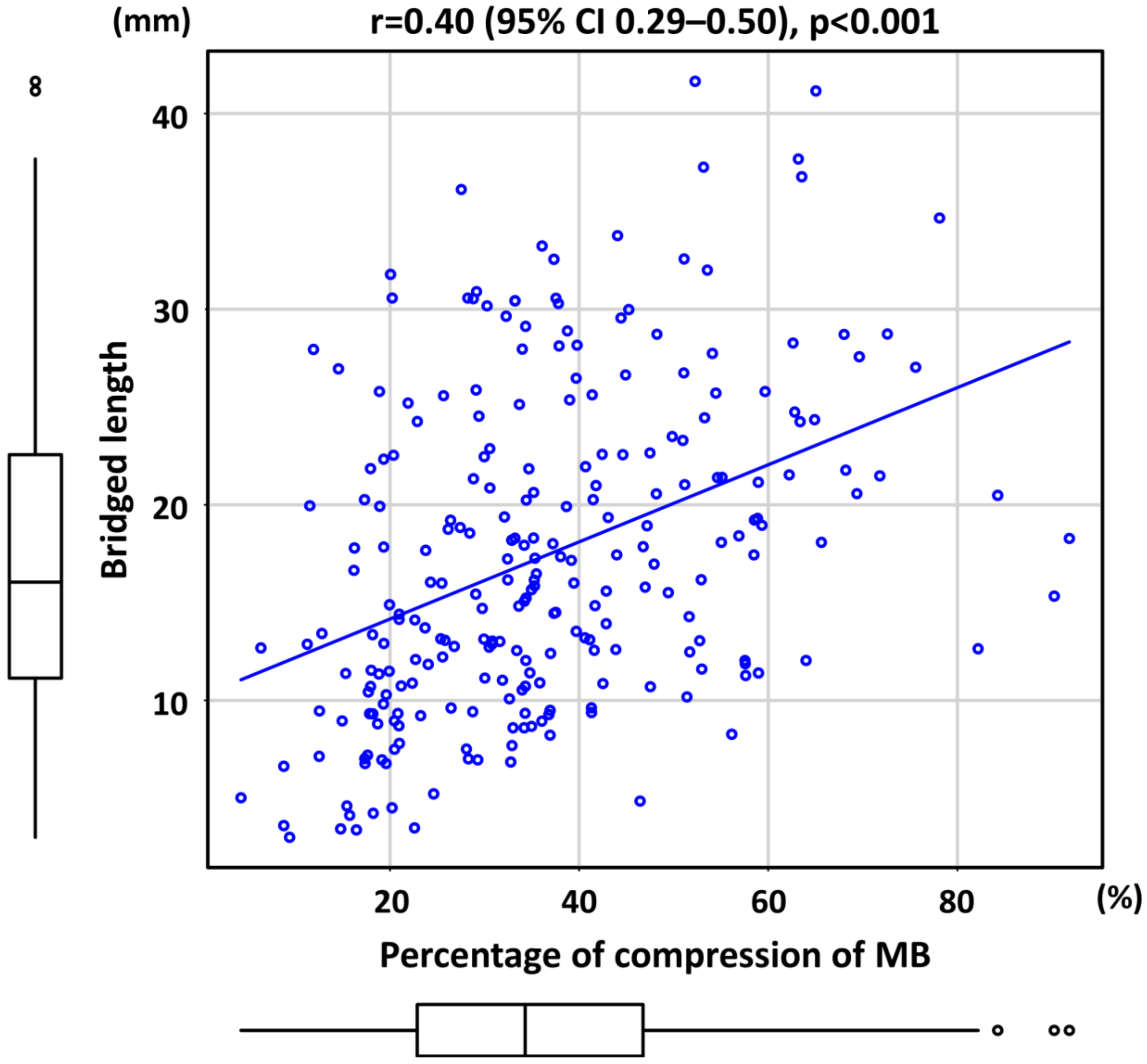

Figure S1. Correlation Between Bridged Length and Percentage of Compression of MB

CI = confidence interval; MB = myocardial bridge.

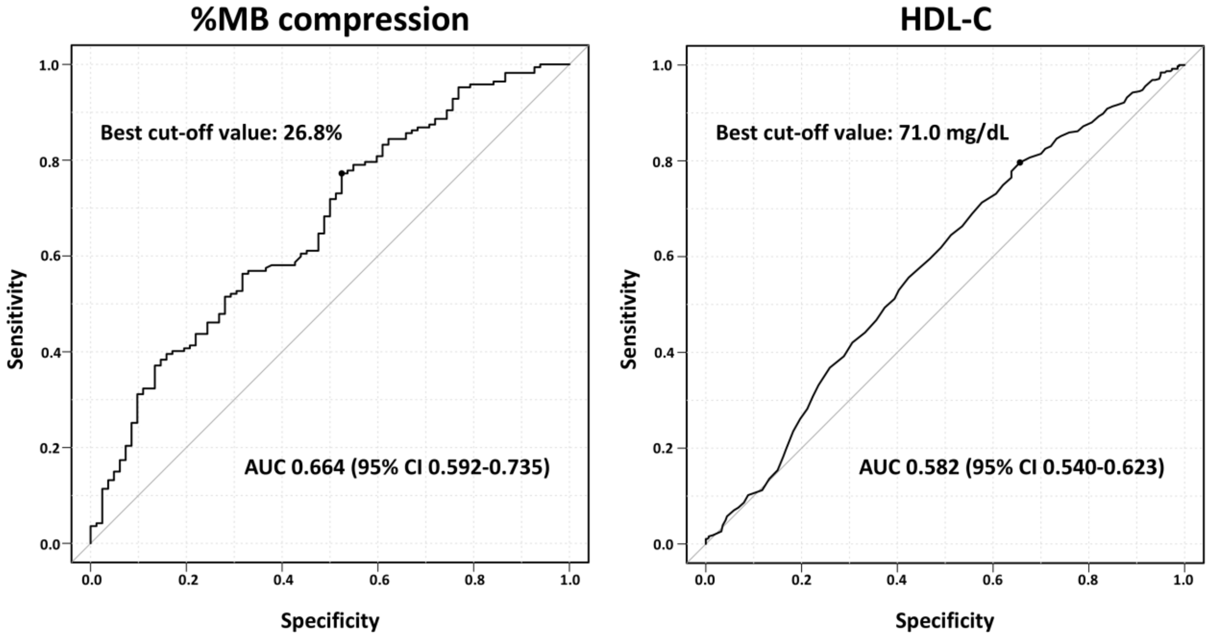

Figure S2. Receiver Operating Characteristics Curve Analysis of HDL-C and Percentage of MB Compression for Positive Acetylcholine Provocation Tests

AUC = area under the curve; CI = confidence interval; HDL-C = high-density lipoprotein cholesterol; MB = myocardial bridge.

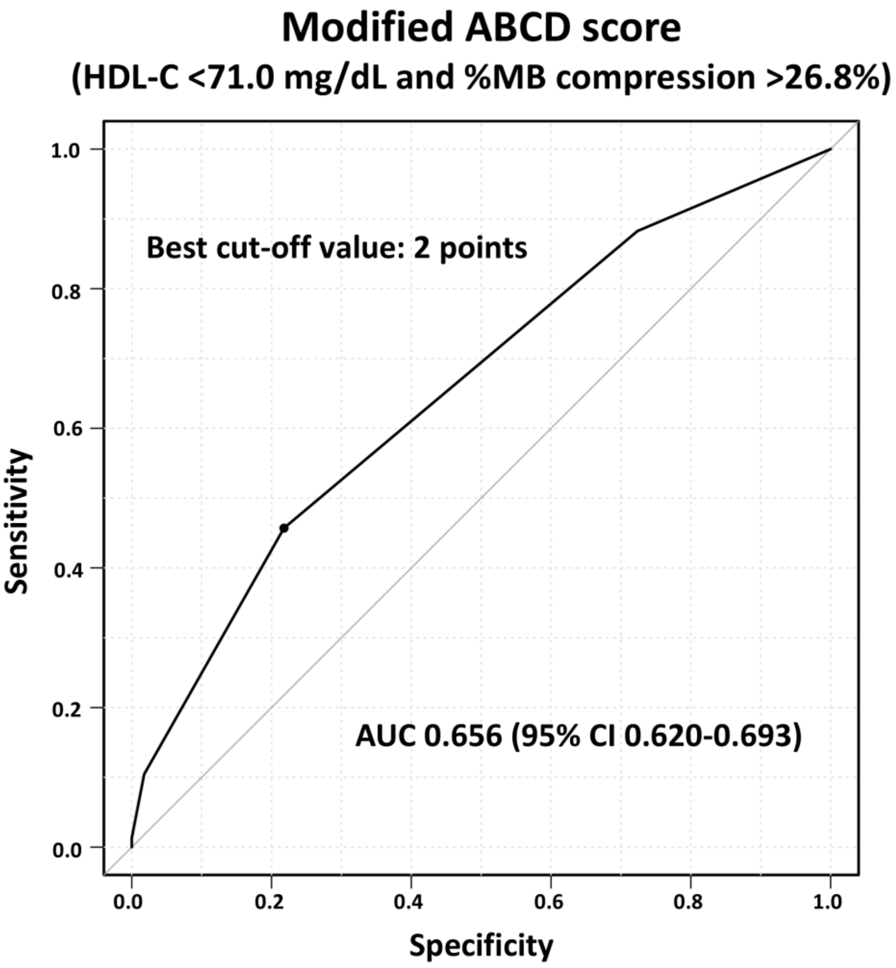

Figure S3. Receiver Operating Characteristics Curve Analysis for Positive Acetylcholine Provocation Test Results

In the modified ABCD score, the best cut-off values of HDL-C (<71 mg/dL) and the percentage of MB compression (>26.8%) on the receiver operating characteristic curve analysis rather than the approximate numbers (70 mg/dL and 30%) were used as a component.

CI = confidence interval; DS = diameter stenosis; HDL-C = high-density lipoprotein cholesterol; LDL-C = low-density lipoprotein cholesterol; MB = myocardial bridge; TG = triglyceride.

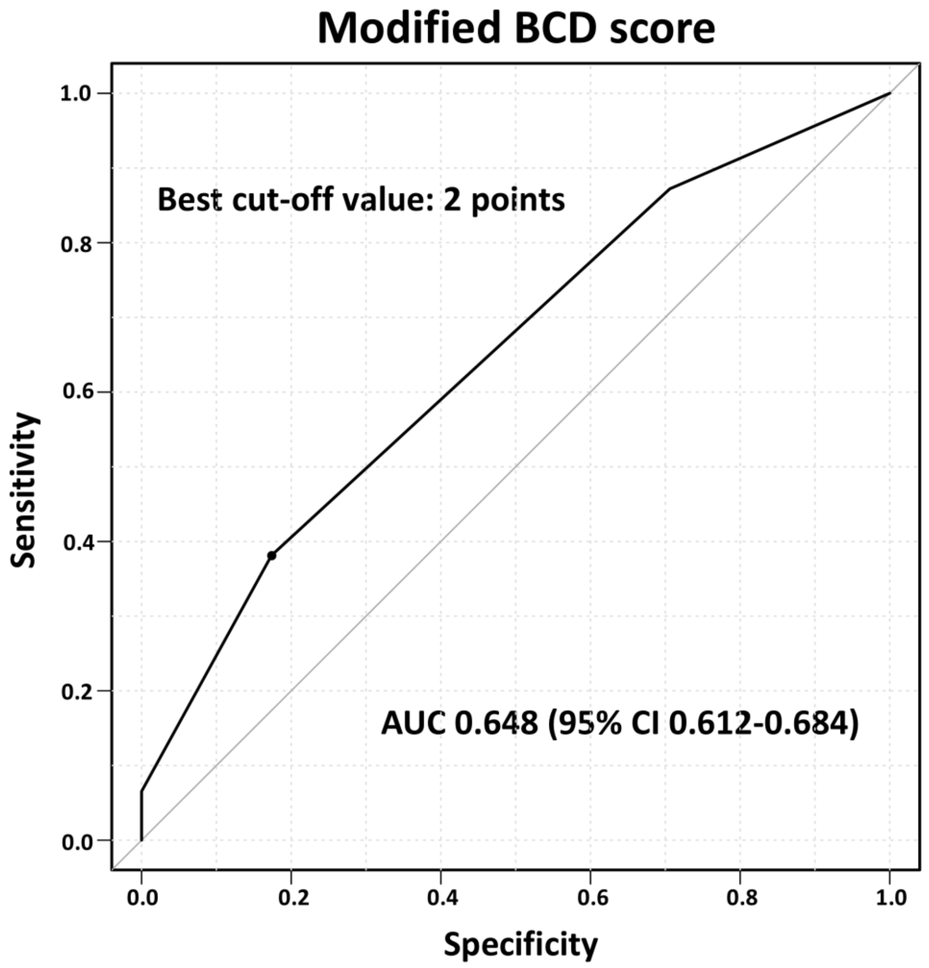

Figure S4. Receiver Operating Characteristics Curve Analysis of the Modified BCD Score for Positive Acetylcholine Provocation Tests

Acute clinical presentation (positive troponin) is omitted from the modified ABCD score.

AUC = area under the curve; CI = confidence interval.

Table S1. Multivariable Logistic Regression Analysis for Positive Acetylcholine Testing

Variable	OR (95% CI)	p value
Men	1.210 (0.879-1.670)	0.241
Dyslipidemia	0.916 (0.658-1.280)	0.605
Current cigarette smoking	2.010 (1.320-3.070)	0.001
MINOCA presentation	1.350 (0.769-2.370)	0.296
HDL-C <70 mg/dL	1.890 (1.330-2.690)	<0.001
CRP >5 mg/L	0.992 (0.568-1.730)	0.977
Presence of MB	2.480 (1.780-3.460)	<0.001

CI = confidence interval; CRP = C-reactive protein; HDL-C = high-density lipoprotein cholesterol; MB = myocardial bridge; MINOCA = myocardial infarction with non-obstructive coronary arteries; OR = odds ratio.

Table S2. AUCs of the ABCD Scores Before and After Optimism Adjustment

Models and components	AUC (95% CI)	
	Before adjustment	After adjustment
Model 1	0.674 (0.635-0.712)	0.669 (0.632-0.706)
MINOCA presentation		
DS of MB (%)		
Current cigarette smoking		
HDL-C (mg/dL)		
Model 2	0.656 (0.620-0.693)	0.656 (0.621-0.692)
MINOCA presentation		
DS of MB >26.8%		
Current cigarette smoking		
HDL-C <71 mg/dL		
Model 3	0.650 (0.613-0.686)	0.649 (0.615-0.685)
MINOCA presentation		
DS of MB >30%		
Current cigarette smoking		
HDL-C <70 mg/dL		

The diagnostic ability of each model of the ABCD scores was evaluated with AUCs on receiver operating characteristics curve analysis for positive acetylcholine rest results. To account for potential overfitting, internal validation using Harrell's bias correction method was performed to adjust optimism with 10000 bootstrap samples. Model 3 indicates the modified ABCD score in the main document.

AUC = area under the curve; CI = confidence interval; DS = diameter stenosis; HDL-C = high-density lipoprotein cholesterol; MB = myocardial bridge; MINOCA = myocardial infarction with non-obstructive coronary arteries.
